# Letter to the Editor: Do Not Forget to Measure Grip Strength

**DOI:** 10.5435/JAAOSGlobal-D-20-00196

**Published:** 2021-02-02

**Authors:** Graham C. Ives, Stuart H. Kuschner

**Affiliations:** Los Angeles, CA

T*o the Editor:* In their recent article, Mbagwu et al^1^ describe low albumin as a risk factor for postoperative complications after total joint arthroplasty. They recommend incorporation of albumin levels in the preoperative workup. In their view, low albumin levels are a marker of malnutrition.

There is another valuable tool which can provide helpful information regarding the overall health of the patient and which can foreshadow potential postoperative problems; a tool which has received notable attention in the orthopaedic literature: grip strength measurement.

Bohannon^2^ reported that “while the literature is not fully consistent, it tends to support grip strength as a predictor of post-operative complications, mortality, and functional decline.” In a subsequent study, Bohannon^3^ reported that “low grip strength was shown consistently to be associated with a greater likelihood of premature mortality, the development of disability, and an increased risk of complications or prolonged stay after hospitalization or surgery.”

Selakovic et al^4^ studied grip strength in hip fracture patients and found that those with weak grip strength “had significantly poorer functional recovery after 3 and 6 months compared to patients with a grip strength above the cutoff points.” In a recent study, Hashimoto, et al. found that preoperative grip strength was a predictor of stair ascent and descent ability after total knee arthroplasty in female patients.^5^ Meessen et al^6^ noted a positive association between hand grip strength and patient-reported outcomes measures after total hip and knee arthroplasty.

Grip strength has been called “an indispensable biomarker.”^7^ As noted by Kumar et al,^8^ “this simple test may be highly beneficial preoperatively in identifying those patients likely to require longer inpatient stay and therefore those who would benefit from early nutritional intervention and focused physiotherapy.”

As with preoperative albumin level, grip strength measurement can be a very useful metric. It is a simple, straightforward test with predictive value.
